# Assessment of Knowledge on Dietary Management of Chronic Kidney Disease Among Patients Undergoing Hemodialysis at a Tertiary Care Hospital in South India: A Cross-Sectional Analytical Study

**DOI:** 10.7759/cureus.55342

**Published:** 2024-03-01

**Authors:** Kannan Shanmugapriya, S Yuvaraj, D Vishnupriya, K Vinitha, G Vijayanila, T Zamrun Begam, M Veeralakshmi, V Thilagavathi, R Vejaiyan, R Thanasekar

**Affiliations:** 1 Medical-Surgical Nursing, College of Nursing, Jawaharlal Institute of Postgraduate Medical Education and Research (JIPMER), Puducherry, IND; 2 College of Nursing, Jawaharlal Institute of Postgraduate Medical Education and Research (JIPMER), Puducherry, IND

**Keywords:** cross-sectional study., south india, hemodialysis, chronic kidney disease, diet, knowledge assessment

## Abstract

Background

This cross-sectional analytical study aimed to assess the level of knowledge on dietary management of chronic kidney disease (CKD) among patients undergoing hemodialysis in a tertiary care hospital in Puducherry, South India.

Methodology

The study was conducted among 86 inpatients diagnosed with CKD and undergoing hemodialysis in the dialysis unit. They were selected by simple random sampling. The self-administered, validated, self-structured questionnaire was used to collect the data. The study was conducted from May to September 2019. Descriptive statistics (frequency, percentage, mean, and standard deviation) and inferential statistics (chi-square) were used to find out the relationship between the level of knowledge and background variables using IBM SPSS Statistics for Windows, Version 25.0 (Released 2017; IBM Corp., Armonk, New York, United States).

Results

The findings indicated that the majority of patients were in the 20-30 age range (36, 41.9%), male (58, 67.4%), from nuclear families (58, 66.3%), with mixed dietary habits (60, 69.8%), and undergoing thrice-weekly hemodialysis (34, 53.5%). Additionally, 59 (68.6%) were hypertensive and 14 (16.3%) were diabetic. Most patients exhibited a moderate level of knowledge (74, 86%), while a small percentage had inadequate (6, 7%) and adequate (6, 7%) knowledge, with a mean (SD) value of 2.00 (0.376). The study identified statistically significant associations between knowledge levels and age, occupation, food habits, duration of dialysis, pre-existing co-morbid illnesses, and treatment of hemodialysis with a p-value <0.05.

Conclusions

In conclusion, this study highlights that the majority of the CKD patients undergoing hemodialysis exhibit moderately adequate knowledge of dietary management. However, a notable need remains for further education and support in this area. Addressing these knowledge gaps is crucial, as it can empower nursing students and healthcare professionals to educate these patients on their dietary needs effectively. By providing comprehensive education and support, we can enhance the quality of care and improve outcomes for hemodialysis patients.

## Introduction

Chronic kidney disease (CKD) is a global health concern affecting approximately 11-13% of the world population across various severity stages. End-stage renal disease (ESRD) is a chronic and potentially fatal health condition in which the kidneys are permanently damaged and the person can no longer survive independently without renal replacement therapy (RRT). RRT involves either dialysis or a kidney transplant. Peritoneal dialysis (PD) and hemodialysis (HD) are the two main forms of dialysis [1]. In India, HD is the preferred option used by most centers. Presently, it is estimated that more than 2.5 lakh people develop kidney failure and one lakh new patients with ESRD enter RRT annually in India [2].

The prevalence of ESRD is anticipated to rise in the United States until 2030, attributed to demographic changes, evolving clinical factors, lifestyle shifts, and advancements in RRT [3]. A very conservative estimate of the ESRD burden, based on a population of 1.1 billion, is that 1,650,000-2,200,000 people develop ESRD in India annually, of whom only 10% or less receive treatment. The rest die every year, which means that approximately 150,000-200,000 people die of ESRD in India annually. The prevalence of CKD and the need for dialysis in India are expected to reach two crores and twenty lakhs, respectively, by 2025 [4]. In 2010, approximately 2.62 million individuals globally underwent dialysis, with projections indicating a doubling of the demand for dialysis by 2030 [5].

Sufficient nutrition is crucial for alleviating the impact of CKD. Low-income groups, often residing in areas with limited access to nutritious foods, face challenges. Implementing population-level strategies such as public education on healthy food choices, regulating fat, salt, and sugar in food, and overseeing programs for public or school meals can significantly enhance kidney health [6]. This CKD manifests with nutritional and metabolic disturbances, particularly in advanced stages. Severe CKD is associated with metabolic issues, chronic comorbidities, and the effects of RRT, such as dialysis, which collectively contribute to negative impacts on nutritional status. Notably, factors such as acidosis, inflammation, oxidative stress, and impaired insulin/insulin-like growth factor axis function can lead to muscle wasting by disrupting the balance between muscle protein anabolism and catabolism [7].

The dietary factor is crucial in managing ESRD, and even a small change in the consumption of any dietary element can greatly influence the development of the condition. Although there have been significant advancements in the science and technology of RRT, the mortality rate among ESRD patients continues to be elevated. Dietary interventions play a crucial role in managing kidney diseases, and nutritional guidance varies based on factors such as the patient's disease stage, the underlying cause, prescribed medications, and other treatment modalities [8].

Dietary therapy is a crucial aspect of treating CKD patients undergoing HD. A kidney-friendly diet can contribute to safeguarding the kidneys from further harm. In the early stages of CKD, adopting a healthy diet may potentially slow the decline in glomerular filtration rate (GFR) and reduce the risk of progressing to complete kidney failure. Individuals with kidney damage should restrict the consumption of specific foods to mitigate the accumulation of metabolic byproducts and provide protection against hypertension, proteinuria, and other cardiovascular and skeletal health issues. Despite extensive research on the impact of certain nutrients on kidney function and the overall health of CKD patients, there is limited literature on how specific dietary choices affect their survival [9]. Helping patients with ESRD attain or maintain the best nutritional status possible is difficult. Even for patients who have been on dialysis for years, maintaining the necessary balance of various nutrients is arduous. The presence of any other chronic condition just amplifies the challenge. Approximately one-third of patients on dialysis have ESRD secondary to diabetic nephropathy [10].

Dietary restriction is also vital for maintaining optimal health in CKD patients because certain substances, such as potassium, sodium, and phosphorous, present in foods and drinks can damage the kidneys when consumed in excess. If these harmful substances are not removed from our body, they can lead to serious complications. So, foods and drinks containing those substances must be regulated by proper excretion [11]. Despite receiving regular dietary instructions, both PD and HD patients exhibit poor knowledge of dietary phosphorus content compared to other essential nutrients for managing CKD [12]. Renin-angiotensin-aldosterone system (RAAS) blockers, though effective for CKD, pose a risk of hyperkalemia, compounded by CKD-related potassium excretion decline. Hyperkalemia, which is life-threatening and linked to adverse outcomes, often necessitates RAAS blocker restriction and low-potassium diets, potentially impacting survival [13]. Over the past decade, a significant advancement in the treatment of chronic renal disease has been the implementation of dietary protein restriction. While altering food intake may not directly enhance kidney function, it plays a crucial role in lessening the burden on the kidneys and enhancing the quality of life for CKD patients undergoing HD. Therefore, it is imperative to evaluate the understanding of dietary management in CKD patients receiving HD [14].

## Materials and methods

A cross-sectional analytical research design and quantitative research approach were used. The objectives are to assess the level of knowledge on dietary management among CKD patients undergoing HD and to identify the association between the knowledge of dietary management and selected background variables. The study was conducted in a tertiary care hospital in Puducherry, South India. The population of the study was the patients who were diagnosed with CKD and undergoing HD in the Dialysis unit as inpatients. The patients diagnosed with CKD and stable undergoing HD for ≥3 months and able to read or speak English or Tamil (local language) were included in this study. The patients who are on peritoneal dialysis and are acutely ill/unstable, such as those with arrhythmias, intradialytic hypo or hypertension, dialysis disequilibrium syndrome, bleeding, seizure, or air embolism, were excluded from the study. The sample size was calculated from the previous study [4], with a confidence interval of 95% and an alpha error of 5% (Results from OpenEpi, Version 3). A sample size of 86 was chosen using simple random sampling with a random table.

Instrument and score interpretation

It consists of two sections. Section I consisted of demographic variables such as age, gender, marital status, religion, domicile, type of family, education, occupation, monthly income, food habits, duration of dialysis, treatment of HD, and pre-existing comorbid illness of HD patients. Section II consisted of 18 multiple-choice questions about the assessment of the level of dietary knowledge regarding CKD patients undergoing HD. Each multiple-choice question has four options with one correct answer. For each correct answer, a score of 1 was given and the wrong answers were given a score of 0. The percentage of the total score obtained by each subject is calculated and classified as follows: scores 0-6, inadequate knowledge; 7-12, moderately adequate knowledge; and 13-18, adequate knowledge.

Reliability and validity of the instrument

The reliability of Cronbach’s alpha for knowledge was 0.82 by the test-retest method. The content of the questionnaire has been validated by experts in the fields of Nephrology, Dietician, Nursing Superintendent, Nursing faculty, and other related fields such as experts from English and Tamil languages for tool translation and back translation. The suggestions and recommendations of the experts were considered.

Data collection procedure and ethical consideration

The study was carried out after approval from the hospital authority, the head of the department of nephrology, and the Nursing superintendent of the dialysis ward. The tool was validated by experts, and ethical clearance was obtained to conduct the study. The responses from the participants were directly collected in the dialysis unit during the dialysis procedure. The participants were asked to be seated in separate places, introduce themselves, and listen to the complaints of the patients and allowed them to vent their feelings, clarify their doubts, and establish a good rapport. The data were collected by a self-administered questionnaire from each patient for 20-30 minutes. The patients who were not able to tick the responses in the questionnaire due to arteriovenous (AV) fistula in the dominant hand were allowed to get help from the researchers. The data were collected from May to September 2019. Descriptive statistics (frequency, percentage, mean, and standard deviation) and inferential statistics (chi-square) were used to find out the relationship between the level of knowledge and variables with a p-value <0.05, which was statistically significant. The data were analyzed using IBM SPSS Statistics for Windows, Version 25.0 (Released 2017; IBM Corp., Armonk, New York, United States).

Ethical clearance was obtained from the Nursing Research Monitoring Committee (NRMC) with the registration number of the proposal JIP/CON/NRMC/B.Sc./2017/GP-X and from the Institute Ethical Committee (IEC) Human Studies with reference number JIP/IEC/2017/0495. Written informed consent was obtained after explaining the purpose of the study to the participants. The patients who experienced discomfort related to the study were given the choice to leave without stating any reason. The confidentiality of the identification details of the study participants was maintained.

## Results

Table 1 shows the frequency and percentage-wise distribution of selected demographic variables among patients undergoing HD. Age distribution indicated a predominant presence in the 20-35 years age group (36, 41.9%), followed by 36-45 years (17, 19.8%), 46-55 years (21, 24.3%), and 56-65 years (12, 14%). Gender distribution highlighted a majority of males (58, 67.4%) compared to females (28, 32.6%). In terms of marital status, 54 (62.8%) were married, while 32 (37.2%) were single. Religious affiliation primarily comprised 69 Hindus (80.2%), followed by 10 Christians (11.7%) and 7 Muslims (8.1%). Domicile analysis revealed a near-equal split between 44 urban (51.2%) and 42 rural (48.8%) residents. The majority belonged to nuclear families (58, 66.3%) as opposed to joint families (28, 33.7%). Educational backgrounds varied, with a significant portion being completed higher secondary (29, 33.7%) and graduation (25, 29.1%). Occupationally, 43 (50%) were unemployed, while 24 (27.9%) held other jobs. Monthly income distribution showed that 62 individuals (72.1%) had an income below Rs. 5000. Treatment frequency revealed that 46 individuals (53.5%) had dialysis twice a week. Pre-existing comorbid illnesses included hypertension (59, 68.6%), diabetes mellitus (14, 16.3%), cardiovascular disorders (2, 2.3%), and other conditions (11, 12.8%).

**Table 1:** Frequency and percentage distribution of demographic and background variables among HD patients (N=86). HD: hemodialysis.

Demographic and background variables	Frequency	Percentage
	(n)	(%)
Age (years)		
20-35	36	41.9
36-45	17	19.8
46-45	21	24.3
56-65	12	14
Gender		
Male	58	67.4
Female	28	32.6
Marital status		
Single	32	37.2
Married	54	62.8
Religion		
Hindu	69	80.2
Christian	10	11.7
Muslim	7	8.1
Domicile		
Rural	42	48.8
Urban	44	51.2
Type of family		
Joint family	28	33.7
Nuclear family	58	66.3
Education		
Illiterate	6	7
Primary	26	30.2
Higher secondary	29	33.7
Graduate	25	29.1
Occupation		
Homemaker	9	10.5
Unemployed	43	50
Skilled	8	9.3
Business	2	2.3
Others	24	27.9
Monthly income		
<5000	62	72.1
5001-10000	10	11.6
10001-15000	6	7
>15001	8	9.3
Treatment of HD		
Once a week	6	7
Twice a week	46	53.5
Thrice a week	34	39.5
Pre-existing comorbid illness		
Diabetes mellitus	14	16.3
Hypertension	59	68.6
Cardiovascular disease	2	2.3
Others	11	12.8

**Figure 1:**
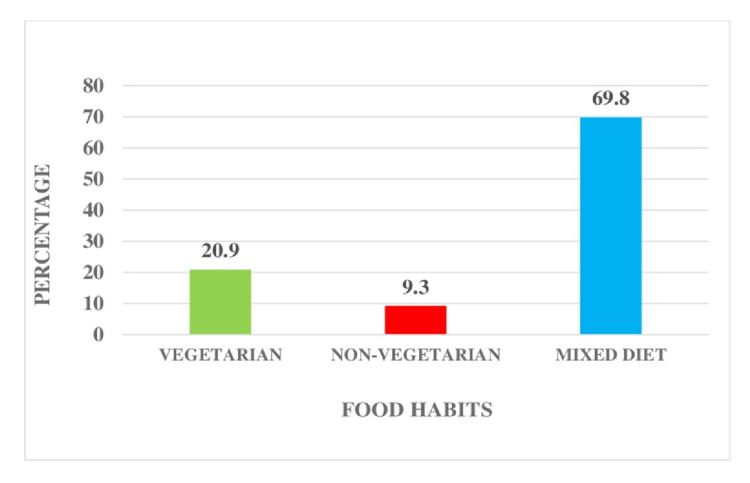
Percentage distribution of food habits among patients undergoing HD. HD: hemodialysis.

Figure 1 shows the percentage distribution of food habits among patients undergoing HD. The majority of patients (60, 69.8%) follow a mixed diet. A notable portion, accounting for 18 patients (20.9%), follow a vegetarian diet, while eight patients (9.3%) adhere to a non-vegetarian diet.

**Figure 2:**
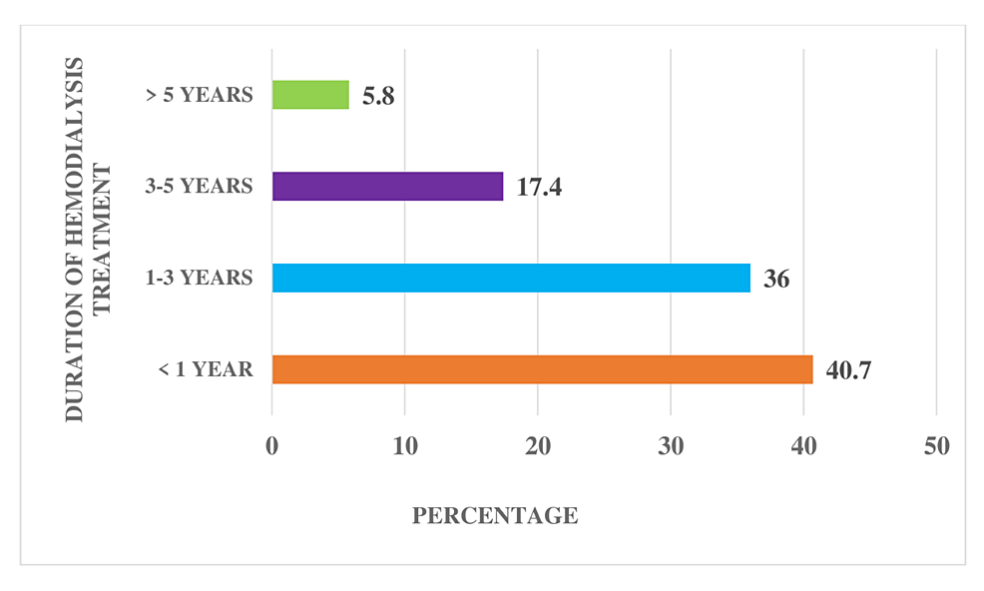
Percentage distribution of the duration of HD treatment among patients undergoing HD. HD: hemodialysis.

Figure 2 shows the percentage distribution of dialysis treatment duration among patients undergoing HD. The distribution of dialysis duration indicates that the majority, 35 (40.7%), have a dialysis history of less than one year. Among them, 31 (36%) fall within the one- to three-year range, while 15 (17.4%) having a dialysis duration spanning three to five years. A smaller subset of five patients (5.8%) have undergone dialysis for over five years.

Table 2 illustrates the distribution of the level of knowledge on dietary management among HD patients. A predominant proportion (74, 86%) of patients exhibited a moderate level of knowledge. Conversely, a minimal number of individuals (6, 7%) undergoing HD demonstrated both inadequate and adequate knowledge levels, with percentages of 6 (7%) each. The mean knowledge level was calculated at 2.00, with a standard deviation of 0.37.

**Table 2:** Level of knowledge on dietary management among HD patients (N=86). HD: hemodialysis.

Level of knowledge	Frequency	Percentage	Mean (SD)
	(n)	(%)	
Adequate knowledge	6	7	2.00 (0.376)
Moderately adequate knowledge	74	86	
Inadequate knowledge	6	7	

Table 3 shows the frequency and percentage distribution of variables and the level of knowledge among HD patients. Among age groups, 33 individuals (91.7%) aged 20-35 exhibit moderately adequate knowledge, while among those aged 56-65, three (25%) have adequate knowledge. Among the genders, 48 males (82.8%) and 26 females (92.9%) possess moderately adequate knowledge. Regarding marital status, 45 married individuals (83.3%) and 29 singles (90.6%) show moderately adequate knowledge, with five married individuals (9.3%) having adequate knowledge. In terms of religion, Hindus constitute 58 (84.1%) of the respondents, with six (8.7%) exhibiting adequate knowledge. Domicile-wise, 39 urban residents (88.6%) and 35 rural residents (83.3%) have moderately adequate knowledge. Family type-wise, 48 (84.2%) respondents from nuclear families and 26 (89.7%) from joint families possess moderately adequate knowledge, with five (8.8%) of nuclear family members having adequate knowledge. Education plays a significant role, as illiterate individuals exhibit moderately adequate knowledge (4, 66.7%), while those with primary, higher secondary, and graduate education show higher percentages of moderately adequate knowledge.

**Table 3:** Frequency and percentage distribution of the level of knowledge among the variables of HD patients (N=86). HD: hemodialysis.

Variables	Level of knowledge					
	Inadequate		Moderately adequate		Adequate	
	n	%	n	%	n	%
Age (years)						
20-35	3	8.3	33	91.7	0	0
36-45	1	5.9	14	82.4	2	11.8
46-55	2	9.5	18	85.7	1	4.8
56-65	0	0	9	75	3	25
Gender						
Male	4	6.9	48	82.8	6	10.3
Female	2	7.1	26	92.9	0	0
Marital status						
Single	2	6.2	29	90.6	1	3.1
Married	4	7.4	45	83.3	5	9.3
Religion						
Hindu	5	7.2	58	84.1	6	8.7
Christian	1	10	9	90	0	0
Muslim	0	0	7	100	0	0
Domicile						
Rural	4	9.5	35	83.3	3	7.1
Urban	2	4.5	39	88.6	3	6.8
Type of family						
Joint family	2	6.9	26	89.7	1	3.4
Nuclear family	4	7	48	84.2	5	8.8
Education						
Illiterate	1	16.7	4	66.7	1	16.7
Primary	0	0	24	92.3	2	7.7
Higher secondary	4	13.8	25	86.2	0	0
Graduate	1	4	21	84	3	12
Occupation						
Homemaker	2	22.2	7	77.8	0	0
Unemployed	1	2.3	39	90.7	3	7
Skilled	0	0	7	87.5	1	12.5
Business	1	50	1	50	0	0
Others	2	8.3	20	83.3	2	8.3
Monthly income						
<5000	4	6.5	54	87.1	4	6.5
5001-10000	1	10	9	90	0	0
10001-15000	1	16.7	4	66.7	1	16.7
>15001	0	0	7	87.5	1	12.5
Food habits						
Vegetarian	1	5.6	15	83.3	2	11.1
Mixed	3	5	53	88.3	4	6.7
Non-vegetarian	2	25	6	75	0	0
Duration of dialysis						
<1 year	2	5.7	29	82.9	4	11.4
1-3 years	4	12.9	26	83.9	1	3.2
4-5 years	0	0	14	93.3	1	6.7
>5 years	0	0	5	100	0	0
Treatment of HD						
Once a week	0	0	5	83.3	1	16.7
Twice a week	3	6.5	39	84.8	4	8.7
Thrice a week	3	8.8	30	88.2	1	2.9
Pre-existing co-morbid illness						
Diabetes mellitus	0	0	12	85.7	2	14.3
Hypertension	5	8.5	51	86.4	3	5.1
Cardiovascular disease	0	0	2	100	0	0
Others	1	9.1	9	81.8	1	9.1

Occupationally, homemakers (7, 77.8%), unemployed individuals (39, 90.7%), and skilled workers (7, 87.5%) predominantly possess moderately adequate knowledge. For instance, among individuals earning less than 5000 monthly, 54 (87.1%) primarily possess moderately adequate knowledge. Regarding diets, vegetarians (15, 88.3%) and those with mixed diets (53, 88.3%) among HD patients showed moderately adequate knowledge. Regarding the duration of dialysis, individuals undergoing treatment for one to three years (26, 83.9%) or less than a year (29, 82.9%) display moderately adequate knowledge. Additionally, 39 (84.8%) patients receiving dialysis twice a week exhibit moderately adequate knowledge. Pre-existing comorbid illnesses contribute to nuanced knowledge levels; among them, 51 individuals (86.4%) with hypertension predominantly possess moderately adequate knowledge. Notably, two persons (100%) with cardiovascular disease exclusively exhibit adequate knowledge.

Table 4 depicts the association between the level of knowledge on dietary management of HD patients and their variables. It was found that age, occupation, food habits, duration of dialysis, treatment of dialysis, and pre-existing comorbid illnesses had a statistically significant association with a p-value <0.05.

**Table 4:** Association between the level of knowledge on dietary management of HD patients and their demographic variables (N=86). Level of significance: *p<0.05; **p<0.01. df: degree of freedom; HD: hemodialysis.

Variables	X^2^	df	p-value
Age	10.2	6	0.009**
Gender	3.12	2	0.210
Marital status	1.24	2	0.536
Religion	2.31	4	0.678
Domicile	.837	2	0.658
Type of family	.847	2	0.655
Education	9.17	6	0.173
Occupation	11.9	8	0.046*
Monthly income	3.69	6	0.718
Food habits	5.28	4	0.019*
Duration of dialysis	6.27	6	0.012*
Treatment of dialysis	16.4	4	0.001**
Pre-existing comorbid illnesses	12.0	6	0.005**

## Discussion

This study aimed to assess the level of knowledge on dietary management of CKD among patients undergoing HD. The majority of the patients undergoing HD had a moderate level of knowledge, and a very smaller number of patients undergoing HD had inadequate and adequate levels of knowledge. This finding is similar to the study conducted in Mangalore, India, on knowledge and practice of dietary management in patients undergoing HD, which concluded that the majority 73.3% had average knowledge, 16.7% of the subjects exhibited good knowledge, and 10% had poor knowledge [15]. It is also similar to the study conducted in Chennai, India, to assess the knowledge, attitude, and practices of renal diets among HD patients, which revealed that 39.7% of HD patients had average to good knowledge of renal diet but did not adhere to the diet pattern [16]. A study conducted in Karwar, Karnataka, India, aimed to assess the knowledge and attitudes of patients undergoing HD regarding their dietary management. The study revealed that the majority (66.6%) had average knowledge, with a mean (SD) value of 8.03 (0.45). Interestingly, the knowledge scores did not show statistically significant differences with any of the demographic variables [17].

There was a statistically significant association with a p-value <0.05 found with the age, occupation, food habits, duration of dialysis, and pre-existing comorbid illness among HD patients. A study conducted in Indonesia concluded that dietary management knowledge among HD patients was statistically significant with their age and duration of dialysis treatment [18]. In Chennai, India, a study was conducted to assess knowledge, attitude, and practice among HD patients on renal diet. It shows that 60.3% possessed excellent knowledge, and there was statistical significance with income and education on renal diet with a p-value <0.001. However, in our study, these variables were not statistically significant [19].

If the educational program focuses on dietary aspects for elderly HD patients, it will yield positive outcomes by enhancing their self-efficacy, elevating dietary knowledge, and fostering improved dietary habits and will reduce the intake levels of sodium, potassium, and phosphorus [20]. However, a descriptive, cross-sectional study conducted in Bloemfontein, South Africa, on the knowledge, attitude, and practices of patients receiving maintenance HD showed that nearly 50% of the HD patients had poor knowledge of dietary restrictions on renal diet, 60% had negative attitudes, and 61.4% had poor practice on diet adherence. Except nutrition education in home language or second language, no demographic variables had statistical significance with dietary knowledge [21].

Patients and caregivers grappling with the complexities of prescription management face conflicting information. Many find it challenging to navigate and accept these prescriptions. Ensuring that family members of dialysis patients provide comprehensive information about the dietary habits and preferences of the patient is crucial. This information aids healthcare providers in tailoring dietary recommendations and interventions to meet the specific needs and challenges faced by individuals undergoing dialysis. Effective communication regarding dietary aspects enhances the overall care and management of dialysis patients, promoting better health outcomes and optimizing the impact of the treatment regimen [22]. A deficient understanding of dietary principles is particularly noticeable among dialysis patients, with even more pronounced challenges observed in individuals with diabetes, hypoalbuminemia, and diminished kidney function and cardiovascular diseases. Timely identification and effective management of symptoms contributing to malnutrition play a crucial role in mitigating mortality risks and slowing the progression of CKD among HD patients [23]. These patients encounter a diverse range of issues, and the nature of their problems can vary widely from person to person. The intervention and dietary counseling are also personalized [24].

Patients on HD typically visit the hospital for dialysis sessions and will engage in consultations with nurses, addressing issues related to self-care behaviors, including dietary restrictions, exercise, medication adherence, and the management of comorbidities and complications. This heightened support from healthcare providers is especially beneficial for individuals with dialysis-dependent CKD, contributing to positive changes in their self-care behaviors [25]. Living with CKD presents challenges for both patients and their care partners. Empowering patients, along with their involved family or friends, can alleviate the burden of CKD symptoms, enabling a more active and engaged life. Shifting the focus toward living well with kidney disease, and emphasizing patient control, is crucial. The World Kidney Day (WKD) Joint Steering Committee designated 2021 as the year of "Living Well with Kidney Disease," aiming to raise awareness and educate on the significance of patient empowerment and active life participation [26].

Improving staffing in renal dietetics is pivotal for patient care, yet it may not enhance access if health professionals don't utilize these services. Raising awareness among them about the evidence supporting dietary management in CKD is vital for consistent patient care and uptake of renal dietetic services [27]. Conventional CKD dietary recommendations often neglect fruit and vegetable consumption, potentially limiting diet diversity. Promoting plant-based diets could offer various benefits, including improved gut health and better control of hyperphosphatemia, with few risks and potential advantages in both CKD prevention and progression [28]. A scoping review conducted in Canada concluded that in HD diet management, mHealth interventions showed promising improvement in patient satisfaction, user acceptance, and reducing healthcare costs, despite varying effects on clinical measurements [29]. Utilize mobile apps and online platforms offering personalized dietary guidance, interactive tools, and educational resources to empower individuals, particularly those with CKD, to improve their dietary knowledge and make informed choices. Additionally, integrate wearable devices and telehealth services to provide real-time feedback and remote support, enhancing accessibility and engagement in dietary management. At the end of the study, nursing students, along with the nursing officers at the dialysis unit, provided health education on dietary management to the patients who had inadequate and moderately adequate knowledge.

The study is limited to individuals diagnosed with CKD and undergoing HD in one setting. These constraints limit the breadth and duration of data gathering as well as the generalizability of findings to a broader population of CKD with varying treatment modalities or treatment options.

## Conclusions

The study reveals that the majority of patients undergoing HD possess a moderate level of knowledge, with only a small fraction exhibiting inadequate and adequate levels of knowledge. Bridging these knowledge gaps is essential for enabling nursing students and nurses to deliver effective education to patients regarding their dietary needs. By addressing these gaps comprehensively, nurses can enhance their capacity to provide accurate and detailed information to patients, empowering them to make informed decisions about their nutritional requirements. This approach not only fosters a stronger nurse-patient relationship but also contributes to improved health outcomes as patients develop a deeper understanding of how dietary choices influence their well-being.
